# BS148 Reduces the Aggressiveness of Metastatic Melanoma via Sigma-2 Receptor Targeting

**DOI:** 10.3390/ijms24119684

**Published:** 2023-06-02

**Authors:** Claudia Sorbi, Silvia Belluti, Claudio Giacinto Atene, Federica Marocchi, Pasquale Linciano, Neena Roy, Elia Paradiso, Livio Casarini, Simone Ronsisvalle, Tommaso Zanocco-Marani, Livio Brasili, Luisa Lanfrancone, Carol Imbriano, Giulia Di Rocco, Silvia Franchini

**Affiliations:** 1 Department of Life Sciences, University of Modena and Reggio Emilia, 41125 Modena, Italy; 2 Hematology Section, Department of Medical and Surgical Sciences, University of Modena and Reggio Emilia, 41124 Modena, Italy; 3 Department of Experimental Oncology, IEO, European Institute of Oncology IRCCS, 20139 Milan, Italy; 4 Department of Drug Sciences, University of Pavia, 27100 Pavia, Italy; 5 Unit of Endocrinology, Department of Biomedical, Metabolic and Neural Sciences, University of Modena and Reggio Emilia, Ospedale di Baggiovara, 41126 Modena, Italy; 6 Center for Genomic Research, University of Modena and Reggio Emilia, 41125 Modena, Italy; 7 Department of Drug and Health Sciences, University of Catania, 95125 Catania, Italy

**Keywords:** sigma-2 receptor, TMEM97, sigma-2 agonist, cancer, melanoma

## Abstract

The management of advanced-stage melanoma is clinically challenging, mainly because of its resistance to the currently available therapies. Therefore, it is important to develop alternative therapeutic strategies. The sigma-2 receptor (S2R) is overexpressed in proliferating tumor cells and represents a promising vulnerability to target. Indeed, we have recently identified a potent S2R modulator (BS148) that is effective in melanoma. To elucidate its mechanism of action, we designed and synthesized a BS148 fluorescent probe that enters SK-MEL-2 melanoma cells as assessed using confocal microscopy analysis. We show that S2R knockdown significantly reduces the anti-proliferative effect induced by BS148 administration, indicating the engagement of S2R in BS148-mediated cytotoxicity. Interestingly, BS148 treatment showed similar molecular effects to S2R RNA interference-mediated knockdown. We demonstrate that BS148 administration activates the endoplasmic reticulum stress response through the upregulation of protein kinase R-like ER kinase (PERK), activating transcription factor 4 (ATF4) genes, and C/EBP homologous protein (CHOP). Furthermore, we show that BS148 treatment downregulates genes related to the cholesterol pathway and activates the MAPK signaling pathway. Finally, we translate our results into patient-derived xenograft (PDX) cells, proving that BS148 treatment reduces melanoma cell viability and migration. These results demonstrate that BS148 is able to inhibit metastatic melanoma cell proliferation and migration through its interaction with the S2R and confirm its role as a promising target to treat cancer.

## 1. Introduction

**Figure 1:**
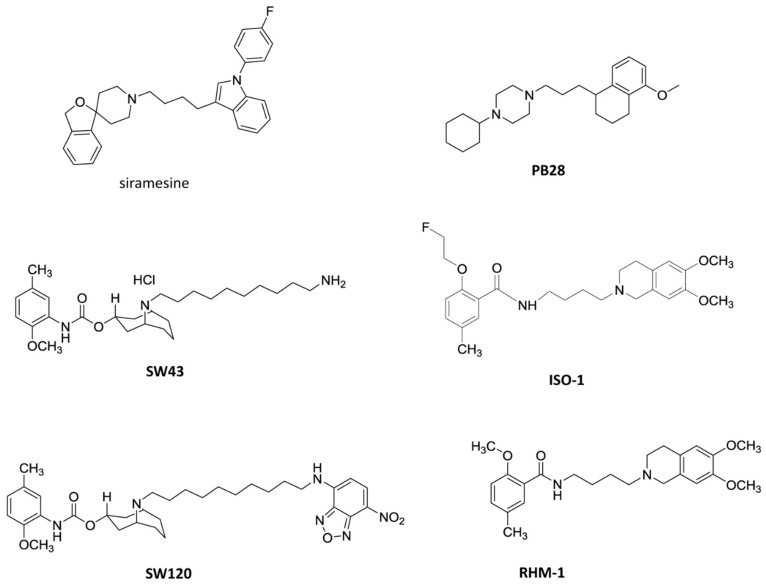
Representative sigma-2 ligands involved in cancer.

Among all types of cancer, cutaneous melanoma is considered one of the most serious because of the high potential for metastasis and mortality worldwide. According to estimates, by 2040, the number of new cases of cutaneous melanoma per year will increase by more than 50% [1]. Although our understanding of the biology of melanoma and the development of targeted and immune therapies have advanced significantly over the past five years, a large fraction of patients do not respond to therapy or rapidly acquire drug resistance [2]. To effectively address this issue and guide future therapy development, a deeper knowledge of the actionable molecular pathways in metastatic melanoma is required and innovative treatments urgently needed. Sigma receptors play an important role in many physiological and pathological conditions, such as brain plasticity, learning and memory processes, CNS disorders, drug abuse, and neuropathic pain [3,4,5,6]. In addition, a growing body of evidence supports their function in tumor development, growth, and migration due to their upregulation in various human cancer cells compared with healthy tissue [7]. Since the identification of the sigma receptors in the mid-1970s, efforts have been made to characterize their molecular structure and function [8,9]. Sigma receptors were historically divided into two distinct subtypes, namely, sigma-1 receptor (S1R) and sigma-2 receptor (S2R), the latter coded by the *TMEM97* gene [10,11]. Crystallization of both S1R and S2R has brought this receptor family into the new era of drug discovery, enabling a more efficient toolbox for the identification of selective molecular probes and new drugs [12,13]. Some representative S2R ligands under investigation for the diagnosis or treatment of cancer are shown in Figure 1. Sigma-1 antagonists and sigma-2 agonists have been reported to induce cell death in several human cancer cells, although there are no sigma-receptor-targeting drugs approved for and currently applied as anti-cancer therapies in clinics [14,15]. Recently, a highly selective sigma-2 radioligand, [^18^F]ISO-1 (Figure 1), has been validated as a PET imaging biomarker to assess the proliferative status of tumors and as a predictor of therapy response using noninvasive techniques [16].

Our research group has been active in the field of sigma-receptor ligands [17]. In our previous works, a large library of substituted benzylpiperidines and benzylpiperazines was synthesized, and the effect of modifications to the aralkyl moiety has been studied systematically [18]. Among them, BS148 exhibited nanomolar affinity and good preference for S2R over S1R (pK*i* S1R = 6.27 (K*i* 540 nM); pK*i* S2R = 7.71 (K*i* 20 nM); S2R/S1R = 28). Functionally, BS148 treatment induced cell death in SK-MEL-2 metastatic malignant melanoma cells with a GI_50_ of 22 μm, which was comparable to the reference S2R agonist siramesine. In the tail-flick test, BS148 decreased the analgesic effect of morphine, supporting an S1R agonist profile and thus excluding the involvement of the S1R in cell death, as S1R agonists show a pro-survival action [14]. Therefore, we assumed that the anti-proliferative effect of BS148 was possibly mediated by the modulation of the S2R, suggesting an S2R agonist profile. Although S2R selective compounds with excellent antitumor activity have been identified, it is still unclear whether their mechanism of action is mediated by this receptor, as opposite results have been obtained [19,20].

To this aim, in this work, we elucidate the molecular basis of BS148 anti-cancer activity by designing and synthesizing a BS148 fluorescent probe for confocal microscopy study. Through siRNA-mediated knockdown of *TMEM97*, coding for S2R, we validated the BS148 target in melanoma cells. In addition, we investigated the effects induced by S2R after treatment with BS148 at the molecular level using Western blotting and RT-qPCRs. Finally, we assessed the anti-proliferative and anti-migration activity of BS148 in metastatic melanoma PDX-derived cells, which represent an important platform for elucidating new treatments in oncology.

## 2. Results

### 2.1. BS148 Enters Melanoma Cells and Localizes into the Cytoplasm

#### 2.1.1. Design and Synthesis of BS148 Fluorescent Probe

**Figure 2:**
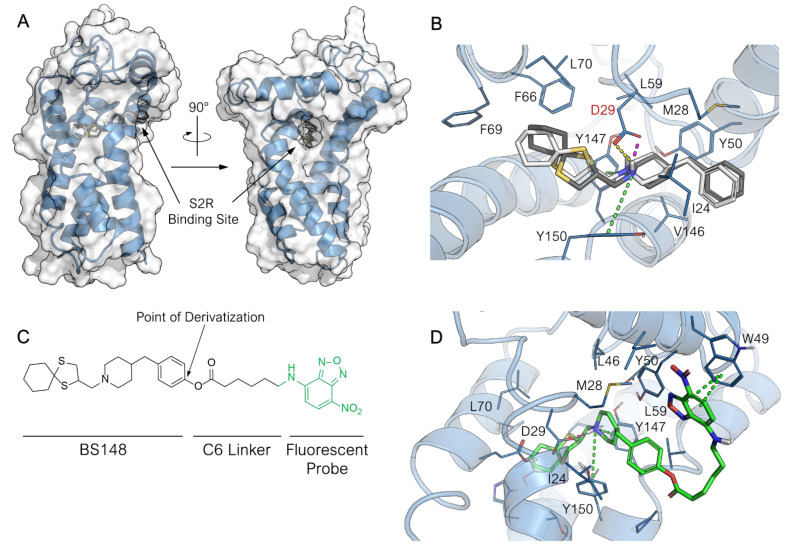
Predicted binding mode of BS148 and BS148-fluo at the S2R binding site. (**A**) Overall structure of S2R in complex with highest-score binding pose of BS148 and view of the entrance to the binding pocket. The tertiary structure of the protein (PDB ID: 7M94) is represented as blue cartoon. The surface of the protein is displayed in transparency. (**B**) Focus on the binding mode of *(S)-*BS148 (in white stick carbons) and *(R)-*BS148 (in grey stick carbon) within the S2R binding pocket. (**C**) Chemical structure of BS148-fluo. The point of derivatization of BS148 for appending the probe is highlighted. (**D**) Binding mode of BS148-fluo (in green stick carbons) with a particular focus on the orientation of the probe over the S2R surface. The 3D structure of S2R is represented in blue cartoon, and the key amino acid residues interacting with the ligand are represented in lines. The heteroatoms are color-coded: oxygen in red, nitrogen in blue, and sulfur in yellow. The H-bonding, salt bridge, π-cation, and π–π stacking are represented in yellow, pink, green, and blue dotted lines, respectively.

To shed light on the mechanism of action of BS148, we first assessed its ability to enter melanoma cells and investigated its subcellular localization using confocal microscopy analysis. To this aim, a BS148 fluorescent probe conjugate (BS148-fluo) was synthesized. A preliminary investigation of the binding mode of BS148 at the S2R binding site was performed with docking calculation to identify the portion of the BS148 molecule more prone to being derivatized with the insertion of the fluorescent probe without significantly affecting the binding of the conjugate to the receptor. The structure of S2R remained a mystery until very recently, when the four X-ray crystallographic structures of bovine S2R in complex with S2R ligands, namely, PB28 (PDB ID: 7M93), roluperidone (PDB ID: 7M94), Z1241145220 (PDB ID: 7M95), and Z4857158944 (K*i* S2R = 4 nM; PDB ID: 7M96), were successfully resolved [13]. In this work, the binary complex S2R-roluperidone (PDB ID 7M94) was used for docking calculation because it is the one with the highest resolution (2.41 Å) among the currently available X-ray crystallographic structures. Molecular docking was performed with AutoDock 4.2.6. The docking model was first validated by redocking the cognate ligand roluperidone into the parent crystal structure. The predicted binding mode for roluperidone closely matched the crystallographic pose with a root-mean-square deviation (RMSD) of 0.95 Å, thus confirming the suitability of the docking protocol for effectively predicting the binding mode of the ligand with S2R. The predicted binding pose for BS148 is reported in Figure 2A. BS148 properly accommodated within the S2R binding site. The binding is primarily driven by the salt bridge between the protonated nitrogen atom of the benzylpiperidine moiety and the key residue Asp29. This electrostatic interaction is known to be crucial for the formation of the biologically active conformation of proteins [21]. Moreover, two additional π-cation interactions between the protonated amine and Tyr147 and Tyr150 contribute to reinforcing the binding of the ligand with the receptor and its positioning within the binding site. Indeed, these polar interactions guide the orientation of the two main hydrophobic moieties of BS148 that flanks the tertiary amine. Specifically, the 1,4-dithiaspiro[4.5]decane moiety is located in the deepest and highly hydrophobic portion of the binding pocket formed by Phe66, Phe69, Leu70, and Tyr150; these hydrophobic interactions contribute to stabilizing the compounds in the binding site. Conversely, the benzyl moiety is oriented toward the entrance of the binding site which is lined with hydrophobic and aromatic residues and opens into the lipidic bilayers of the membrane where S2R is anchored rather than the aqueous environment of the cytoplasm. Interestingly, no significative differences in the binding mode or the docking score were observed for either *R-* or *S-*enantiomers (docking score −8.439 and −8.415 for *(S)-*BS148 and *(R)-*BS148, respectively; Figure 2B).

Based on the predicted binding mode of BS148, we chose the benzyl moiety as the anchor point for the introduction of the fluorescent probe, as it is located in a wider region outside the binding pocket and therefore should not adversely affect the binding of the conjugate to S2R (Figure 2C). We chose the green-emitting 7-nitrobenzofurazan as a fluorogenic moiety previously used in confocal microscopy study for selective S2R fluorescent probes [22]. This fluorescent probe was connected to the functionalized BS148 through a six-carbon spacer. Thus, the binding of BS148-fluo at S2R was preliminarily investigated with docking calculation. No significative differences between BS148-fluo and the parent compound in the binding mode for the core 1-((1,4-dithiaspiro[4.5]decan-2-yl)methyl)-4-benzylpiperidine scaffold were observed. As for BS148, the chirality seemed to also not affect the binding of BS148-fluo (docking score −10.142 and −9.859 for *(S)-*BS148-fluo and *(R)-*BS148-fluo, respectively). As expected, the fluorescent 7-nitrobenzofurazan moiety is completely positioned outside the binding pocket. This results in several predicted orientations for the 6-(7-nitrobenzofuran-4-ylamino)hexanoate moiety. To assess the binding stability of the BS148-fluo binding pose and investigate the preferred and most recurring orientation of the 6-(7-nitrobenzofuran-4-ylamino)hexanoate tail, molecular dynamic simulation was performed. To reproduce a more realistic environment, the S2R was embedded within a membrane bilayer. The positioning of S2R within the membrane was retrieved from the OPM (Orientation of Proteins in Membrane) database. The prepared system was subjected to a 50 ns MD run. To obtain insight into the dynamic behavior and stability of the ligand complexes, the backbone Root-Mean-Square Deviation (RMSD) was computed. The RMSD of the system sharply increased during the initial equilibration phase because of the change in simulation condition but rapidly converged at 1.4 ± 0.2 Å after 2 ns. The protein RMSD does not fluctuate significantly throughout the duration of the simulation (Figure S1A), thus indicating the dynamic stability of the system upon BS148-fluo binding to the receptor. The ligand RMSD was measured as well to assess the stability of the ligand with respect to the protein and its binding pocket. The RMSD of the ligand increased to 3.159 ± 0.445 Å during the initial equilibration phase as a result of the settling of the ligand from its docked position and then underwent a further sharp increase at around 5.2 ns of simulation. The RMSD of the ligand fluctuated around 6.000 ± 1.000 Å for the next 15 ns and then converged at 22 ns and settled to 5.863 ± 0.221 Å for the entire duration of the simulation. To understand the reasons for this change in the RMSD of the ligand, the Ligand Root-Mean-Square Fluctuation (L-RMSF), which expresses the changes in the ligand atom positions, was computed. As displayed in the Ligand RMSF plot reported in Figure S1B, the BS148 core of the conjugate molecule is firmly anchored within the S2R binding site, and it does not show remarkable fluctuation during the entire simulation with an RMSF of 0.895 ± 0.200 Å (atoms 1–25, Figure S1B). Conversely, the 6-(7-nitrobenzofuran-4-ylamino)hexanoate moiety (atoms 26–45) had the highest RMSF of 3.286 ± 0.544 Å, indicating a greater fluctuation for this membrane-exposed portion of the molecule. Considering the average conformation assumed by BS148-fluo during the latest converged 25 ns (Figure 2D), the 7-nitrobenzofurazan moiety folded over the surface of S2R, and it was accommodated within a minor hydrophobic groove on the surface of the protein delimited by helix-1 and -2 and outlined by the hydrophobic amino acid side chains such as Ile 24, Leu27, Met28, Leu43, and Leu46, where in addition to the hydrophobic interactions, an almost conserved π–π interaction was established between the aromatic ring of the benzofurazan moiety and the side chain of Trp49. Summation of the overall frequency of contacts to the binding site of S2R supports the docking results and confirms the capability of BS148-fluo to form stable interactions with the receptor.

**Scheme 1:**
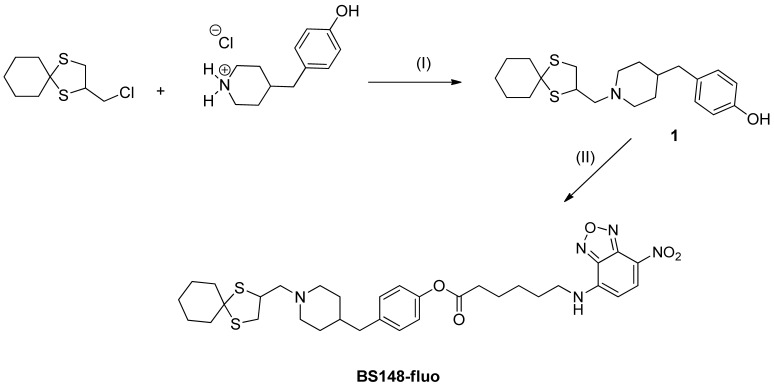
BS148-fluo. Reagents and conditions: (I) anhydrous acetonitrile, K_2_CO_3_ (2 eq.), catalytic KI, 135 °C, MW; (II) 6-(7-nitrobenzofuran-4-ylamino)hexanoic acid, methylene chloride, 1-ethyl-3-(3-dimethylaminopropyl)carbodiimide (EDC), Ar, triethylamine (TEA), r.t.

The synthetic pathway used to obtain the BS148-fluo is depicted in Scheme 1. We planned to insert a hydroxyl group at the para position of the benzyl moiety of BS148 to facilitate bond formation with the selected fluorescent probe 6-(7-nitrobenzofuran-4-ylamino)hexanoic acid. Briefly, the previously synthesized 2-(chloromethyl)-1,4-dithiaspiro[4.5]decane was reacted with piperidin-4-ylmethylphenol (2 eq.) to give the key intermediate 4-((1-((1,4-dithiaspiro[4.5]decane-2-yl)methyl)piperidin-4-yl)methyl)phenol (**1**) [23]. Two microwave (MW) cycles of 3.5 h each were performed at 130–140 °C, affording the desired product in low yield (14%) due to the low nucleophilicity of the amine, commercially available as hydrochloride salt. Compound **1** was then reacted with the 6-(7-nitrobenzofuran-4-ylamino)hexanoic acid using the conventional EDC coupling chemistry (EDC, 1-ethyl-3-(3-dimethylaminopropyl)carbodiimide) in anhydrous methylene chloride (MC) under argon atmosphere in the presence of a catalytic amount of triethylamine, affording the desired compound in 35% yield. BS148-fluo was then converted into the corresponding oxalate salt in order to obtain a water-soluble probe required for the biological tests.

The dose-response curve for BS148-fluo at S2R is reported in Figure S2. As shown in Table 1, the S2R binding affinity of the BS148-fluo is comparable to that of the parent compound BS148. This result agrees with the docking studies.

#### 2.1.2. Confocal Microscopy Studies

**Figure 3:**
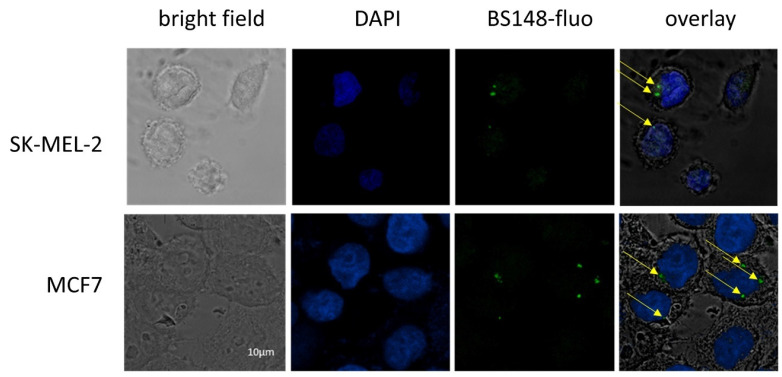
Confocal microscopy analyses of SK-MEL-2 cells labeled with BS148 fluorescent probe. MCF7 cells were used as a positive control. Bright field (first column); DAPI staining (blue, second column); staining with BS148-fluo (100 nM, green, third column); overlay (fourth column). Arrows indicate the localization of BS148-fluo within the cells. Images are representative of one experiment out of three.

BS148-fluo was used to assess BS148 cellular localization using confocal microscopy. SK-MEL-2 cells were grown to sub confluence and treated with BS148-fluo (100 nM), and the fluorescent intensity was analyzed with confocal microscopy. MCF7 cells were used as a positive control, as they express S2R at high levels but low S1R [24]. The results demonstrate the internalization of BS148-fluo in SK-MEL-2 and MCF7 cells (Figure 3). The localization of the probe in the perinuclear region and cytoplasm and the absence of uptake in the nucleus are consistent with the localization of S2R in the endoplasmic reticulum [25].

### 2.2. S2R Is Involved in BS148-Induced Decrease in Cell Viability

**Figure 4:**
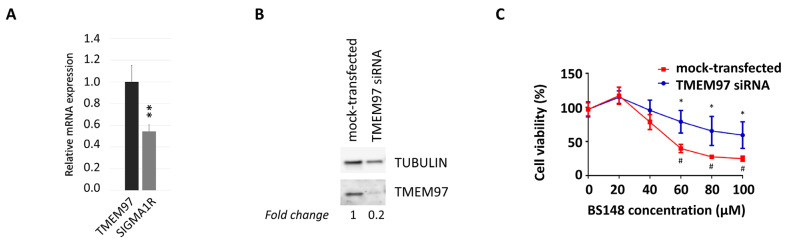
TMEM97-mediated decrease in cell viability induced by BS148. (**A**) qRT-PCR analysis of TMEM97 and S1R transcripts in SK-MEL-2 cells. Normalized S1R mRNA levels are reported as fold change (2-dCT) vs. TMEM97, arbitrarily set at 1 (mean ± SD; n = 3); *t* test was used to calculate statistical significance (** *p* < 0.01). (**B**) Western blot analysis of S2R protein expression in control or TMEM97 knocked down SK-MEL-2 cells. Tubulin served as a loading control. TMEM97 protein levels, derived from densitometry analysis, were normalized to tubulin and expressed as fold change versus mock-transfected control cells. (**C**) The viability of control and knocked-down cells was evaluated with 3-(4,5-dimethylthiazol-2-yl)-2,5-diphenyltetrazolium bromide (MTT) colorimetric assay after 48 h of treatment with 20–100 µM BS148. Cells transfected with control siRNAs served as a positive control (mock). Absorbance data were collected and represented as the percentage (%) of viable cells (means ± SD). * = significantly different versus control in the same treatment condition; # = significantly different versus the untreated mock-transfected condition (two-way ANOVA; *p* ≤ 0.05; n = 8).

The analysis of the expression of sigma receptors in SK-MEL-2 cells, using RT-qPCR, highlighted higher transcript levels of S2R compared to S1R (Figure 4A). We then evaluated whether the anti-proliferative effects induced by BS148 can be ascribed to specific S2R targeting. To this purpose, we knocked down *TMEM97* mRNA transcripts with siRNA delivery in SK-MEL-2 cells and evaluated viability following the administration of increasing concentrations of BS148 for 24 h and 48 h. Western blot analysis of the total cellular extracts confirmed the decrease in S2R protein expression by RNA interference (Figure 4B). The comparison between the growth curves of *TMEM97*-silenced and control (mock-transfected) cells showed significant protection from cell death by *TMEM97* siRNA upon 60–100 µM administration of BS148 (Figure 4C), with the GI_50_ being higher than 100 µM compared to 56.6 ± 22.7 µM in the mock-transfected cells.

### 2.3. BS148 Affects the Expression of Genes Involved in Cholesterol Biosynthesis and Activates the ER Stress Response in Melanoma Cells

**Figure 5:**
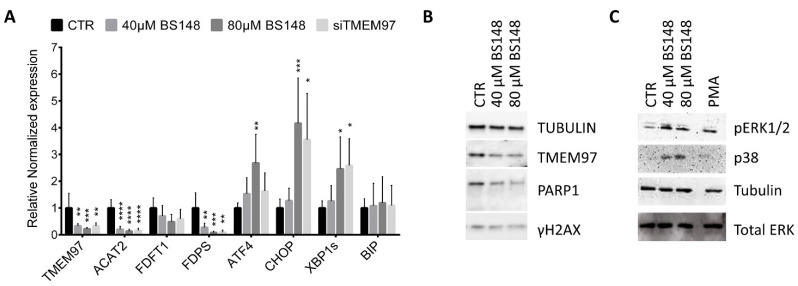
Effects of BS148 treatment in SK-MEL-2 cells. (**A**) RT-qPCR analysis of the indicated transcripts in SK-MEL-2 cells treated with BS148 for 48 h or siRNA against *TMEM97* transcripts. *RPS20* and *GAPDH* were used as reference genes. Normalized mRNA levels are reported as fold change vs. control cells (CTR), arbitrarily set at 1. Data represent mean  ±  SD, n = 6 for BS148 treatments and n = 3 for siTMEM97 transfections; one-way ANOVA was used to calculate statistical significance (* *p* < 0.05, ** *p* < 0.01, *** *p* < 0.001, **** *p* < 0.0001) vs. CTR. (**B**) Western blot of total extracts from SK-MEL-2 cells treated 24 h with BS148 with the indicated antibodies. Tubulin has been used as loading control. (**C**) Representative images of the phosphorylation of ERK1/2 and p38 MAPK using Western blotting. Cells were treated 4 h with 40 µM and 80 µM BS148. Phorbol-12-Myristate-13-Acetate (PMA) was used as a positive control. Total ERK and tubulin were used as normalizer for pERK1/2 and p38 MAPK, respectively.

The membrane-bound S2R localizes in multiple subcellular organelles, including the endoplasmic reticulum (ER), and several lines of evidence highlight the crucial role of S2R in cholesterol homeostasis [26]. With accelerated cholesterol and lipid metabolism being the hallmarks of tumor cells, we hypothesized that BS148 could modulate the expression of genes participating in cholesterol biosynthesis that sustain the proliferation and migration of cancer cells. RT-qPCR analysis on the total RNA extracted from the control and BS148-treated cells at 40 µM and 80 µM doses showed a dose-dependent decrease in the expression of Acetyl-CoA acetyltransferase (Acat2), Farnesyl-diphosphate farnesyltransferase 1 (Fdft1), and Farnesyl diphosphate synthase (Fdps), all genes related to the cholesterol pathway (Figure 5A). Taking into consideration that ER stress regulates cholesterol metabolism and homeostasis, we analyzed the expression of genes related to the ER stress response as well. The transcriptional activation of activating transcription factor 4 (ATF4), C/EBP homologous protein (CHOP), and the short splice variant of the IRE-1α/X-box binding protein 1 (Xbp1) gene support the hypothesis that S2R targeting by BS148 induces ER stress. Differently, the expression of the protein-folding ER chaperone BiP/Grp78/HspA5 that improves the protein-folding function of the ER was not increased. Unexpectedly, the analysis of mRNA levels in the SK-MEL-2 cells knocked down for TMEM97 by RNAi perfectly overlapped with the transcriptional effects induced by BS148 administration. To shed light on this result, we quantified the *TMEM97* mRNA levels following the administration of BS148. RT-qPCR showed a striking decrease in *TMEM97* transcription that was further validated with Western blot protein analysis of the same samples. As shown in Figure 5B, the protein levels were consistent with the mRNA levels. The increased expression of ER stress genes was coherent with the activation of a critical process known as unfolded protein response (UPR) which is usually associated with ER disturbance. At first, the UPR promotes an adaptive mechanism to restore ER homeostasis, but if the stress is prolonged or the adaptive response fails, the cells undergo apoptotic cell death. The analysis of the expression levels of γH2AX and cleaved PARP1 suggests that BS148 administration did not trigger DNA damage and caspase-dependent apoptosis, but rather it could inhibit cell proliferation or caspase-independent cell death (Figure 5B). We further explored ER stress by investigating the related signaling pathways (Figure 5C). The p38 MAPK promotes ER stress-induced cellular processes and may phosphorylate CHOP to activate its transcription through ATF6 [27]. Western blot on the total protein extracts from the BS148-treated cells showed a dose-dependent increase in p38 MAPK phosphorylation. ER stress and UPR were also coupled with the MEK/ERK signaling pathway. Similarly, we observed the phosphorylation of ERK1/2, which could contribute to non-caspase-dependent pathways of injury after ER stress [28].

Overall, these results suggest that the inhibition of the cholesterol pathway associated with the activation of the ER stress response could be responsible for the anti-proliferative activity induced by BS148.

### 2.4. BS148 Reduces Viability and Migratory Capacity of Metastatic Melanoma PDX Cells

**Figure 6:**
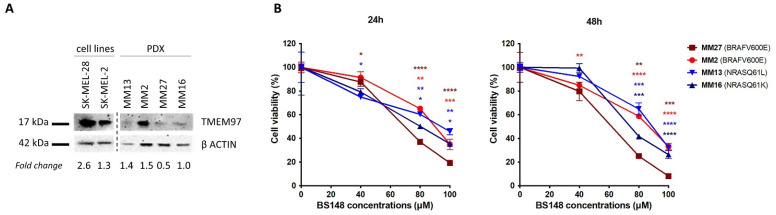
Effects of BS148 treatment in PDX cells. (**A**) Expression of S2R in SK-MEL-2, SK-MEL-28, and metastatic melanoma PDX-derived cells with Western blotting. TMEM97 protein levels, derived from densitometry analysis, were normalized to β-actin and expressed as fold change versus MM16. (**B**) Cell Titer Glo Assay for viability assessment upon BS148 treatment for 24 h (left) and 48 h (right). Cell viability is reduced by increasing concentrations of BS148 in the four PDXs. Viability was normalized to CTR (DMSO-treated cells); triplicate experiment (n = 3); one-way ANOVA was used to calculate statistical significance (* *p* < 0.05, ** *p* < 0.01, *** *p* < 0.001, **** *p* < 0.0001) vs. CTR.

The expression of S2R in metastatic melanoma PDX-derived cells (MM13, MM2, MM27, MM16) [29] was investigated using Western blot analysis and compared to that in SK-MEL-2 and SK-MEL-28 (Figure 6). While at different levels, all the PDX cells show S2R expression, regardless of their genetic background (MM13/MM16 are NRAS-mutant and MM2/MM27 BRAF-mutant melanomas) (Figure 6A). Then, we investigated the anti-proliferative activity of BS148 in PDX cells, showing that increasing doses of BS148 significantly reduced cell viability in all four melanomas (Figure 6B).

**Figure 7:**
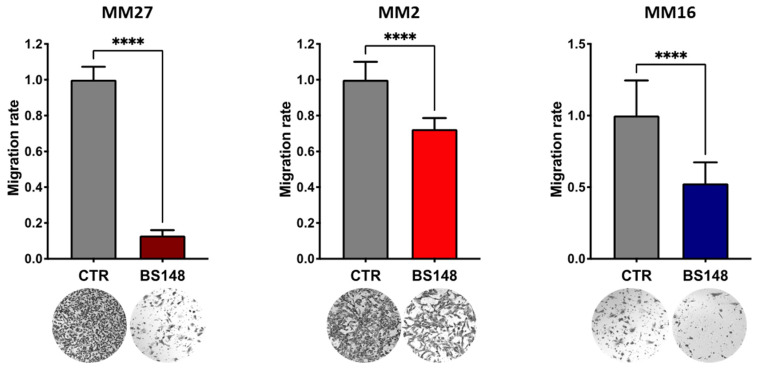
BS148 reduces the migratory properties of PDX cells. PDX cells were pre-treated with 50 μM BS148 for 24 h and then plated in modified Boyden chambers. Cell migration was assessed after 36 h and normalized to CTR (DMSO-treated cells); triplicate experiment (n = 3). *t* test was used to calculate statistical significance (**** *p* < 0.0001) vs. CTR (DMSO-treated).

Moreover, BS148 strongly reduces the migratory features of three PDXs (Figure 7).

**Figure 8:**
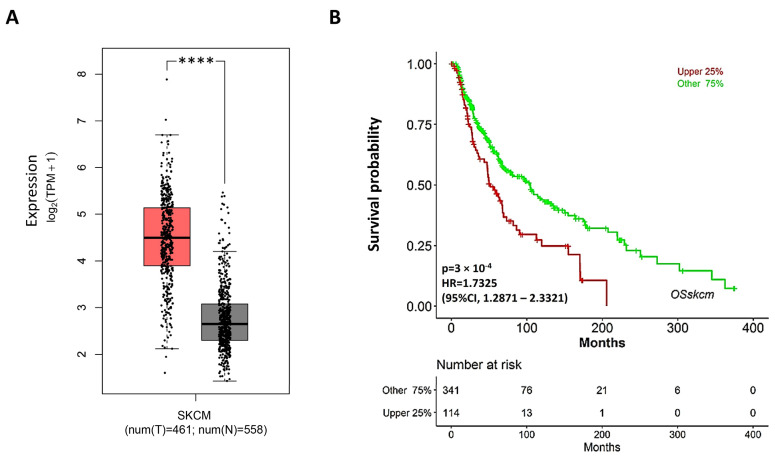
*TMEM97* expression increases in SKCM patients, and its higher levels are associated with poor prognosis. (**A**) Expression levels of *TMEM97* measured as transcripts per million (TPM) in skin cutaneous melanoma (SKCM) patients (red bar, T) compared to normal ones (grey bar, N). **** *p* < 0.0001. (**B**) Overall survival curve of cutaneous melanoma patients based on SKCM TCGA data. Upper 25%: cases ranked in top 25% higher expression level for *TMEM97* gene; other 75%: cases ranked in bottom 75% lower expression level of *TMEM97* gene.

These findings prompted us to evaluate the potential therapeutic relevance of S2R in the management of melanoma patients. Hence, we analyzed RNAseq data from the TCGA (The Cancer Genome Atlas) and GTEx projects to determine the transcript levels of *TMEM97* in patients with melanoma compared to those in healthy patients (Figure 8). *TMEM97* mRNA levels are significantly increased in tumor vs. healthy tissues, supporting the rationale of *TMEM97*-targeting drugs. Moreover, high levels of *TMEM97* expression are associated with patients with poor overall survival probability, hinting that it may be used as a prognostic marker of survival in patients affected by skin cutaneous melanoma (SKCM).

## 3. Discussion

The S2R is an endoplasmic-reticulum-resident transmembrane protein overexpressed in various proliferative tumors. Its expression is positively correlated with tumor progression and recurrence and poor survival [7,11]. Here, we show that *TMEM97* transcript levels rise significantly in tissues from SKCM patients compared to those from healthy ones, and high levels could represent a prognostic marker for the survival prediction of patients. S2R is an interesting pharmacological target because of its involvement in cholesterol homeostasis. Melanoma cells are characterized by an impressive metabolic plasticity, including the lipid metabolic network that confers cancer aggressiveness. The analysis of TCGA data from SKCM patients demonstrates that enhanced expression of cholesterol synthesis genes is associated with decreased melanoma patient survival [30]. These data support the rationale for developing S2R-targeting molecules that could be further developed as anti-cancer drugs. Novel ligands were recently developed to induce cancer-specific cytotoxic activity through the receptor [20], although opposite results were also achieved [19]. In fact, ligands inducing receptor coupling to death pathways are limited, while others mediate effects consistent with cancer cell survival [31]. These diverging effects are due to conformational issues of the receptor in the active state, resulting in intracellular signaling bias and ligand-specific, preferential activation of metabolically stimulative or inhibiting effects.

Our research team has previously identified a sigma ligand, BS148, which exhibited nanomolar affinity and good preference (25-fold) for S2R over S1R, as assessed with receptor-binding assay [18]. This compound showed a marked cytotoxic effect on SK-MEL-2 cells and was classified as a putative S2R agonist on the basis of the commonly accepted definition [32]. In this work, we translated our results into patient-derived xenograft (PDX) cells, proving that BS148 exerts anti-proliferative effects not only in SK-MEL-2 cells but also in melanoma PDX-derived cells. In these cellular models, we also observed inhibitory activity on the migration properties following BS148 treatment. Parallel studies of cell viability in control and knocked-down metastatic melanoma cells demonstrated that the anti-proliferative effects observed following BS148 administration are mediated by S2R targeting, although we cannot exclude that additional molecular targets may be involved. Overall, our results strengthen the concept that S2R could be a candidate for pharmacological anti-cancer treatments [33], including against melanoma [34].

Through the analysis of protein extracts and RNA, we investigated the possible molecular effects at the base of BS148 anti-proliferative activity. With cholesterol homeostasis being one of the main activities ascribed to S2R, we focused on the expression of genes representative of the cholesterol pathway. The expression of the selected genes was previously shown to correlate with S2R activity and expression [35]. The decrease in the transcription of cholesterol genes in BS148-treated cells comparable to *TMEM97* knocked-down cells prompted us to investigate *TMEM97* expression. mRNA and protein levels were similarly reduced in BS148-treated cells, a phenomenon previously described at the protein level with other S2R ligands [20]. Interestingly, the decrease in *TMEM97* expression exerted by BS148 was similar to that observed for S2R RNA interference-mediated knockdown, which has been already shown to inhibit both proliferation and migration of cancer cells [36]. While compounds that mimic S1R knockdown have been historically classified as S1R antagonists, in the case of S2R ligands, the definition of agonist or antagonist activity is still controversial and appears to be context-dependent. Therefore, the negative correlation between BS148’s anti-proliferative effect and TMEM97 expression levels does not hamper the classification of BS148 as an S2R agonist, as was recently shown for PB28, another well-known S2R agonist [37].

The observed decrease in *TMEM97* expression induced by BS148 can even represent an advantage in terms of therapeutic application, as small molecules are extremely stable, easier to administer, and less expensive compared to genetic manipulation. Various mechanisms could be involved in BS148-mediated *TMEM97* transcriptional regulation. For example, TMEM97 has been identified as a target of the oncosuppressor p53 [38] or of c-Jun NH(2)-terminal kinase 2 (JNK) [39] and TGF-ß1 [40]. Further molecular analysis would better identify whether BS148 can affect the expression of these regulators that, in turn, could modulate TMEM97 transcription. Moreover, because *TMEM97* itself is regulated by cholesterol-related signals, such as sterol depletion or SREBP expression levels [26], it could be that BS148 activates a feedback loop that impairs the expression of cholesterol proteins and consequently affects *TMEM97* gene expression. Finally, we cannot exclude that BS148 triggers a desensitization-like mechanism that leads to the decrease in TMEM gene expression. Cholesterol homeostasis and ER stress are highly connected, and altered cholesterol metabolism is a well-documented inducer of ER stress [41]. Correspondingly, we observed a concomitant overexpression of ER stress genes that hinted at the activation of the IRE1 and PERK pathways [42]. Further, Western blot analysis displayed both pERK1/2 and p38 MAPK activation as the main intracellular signaling signature. The activation of pERK1/2 is even slightly enhanced upon cell treatment with 40 µM BS148, while it is not at higher concentrations, suggesting a biphasic behavior of the compound. Moreover, we described for the first time the link between S2R and the p38 MAPK pathway, which occurs under treatment with BS148 and extends the phosphorylation landscape of the receptor to a new partner. The role of p38 MAPK was previously investigated in melanoma, where this kinase may exert inhibitory effects on cancer cell growth and could be targeted to block its proliferation [43,44,45,46,47,48,49,50,51]. Opposite results were also found [52,53,54], demonstrating that the analysis of one single signaling pathway could not provide a comprehensive explanation of the whole metabolic pattern of the cell and that further investigations are required to achieve solid conclusions. For instance, opposite effects on melanoma cell metabolism could be supported by different p38 MAPK isoforms not evaluated herein [55]. In any case, the activation of this kinase may upregulate p53, plausibly via reactive oxygen species (ROS) generation, leading to melanoma cell apoptosis [55,56,57,58]. Interestingly, the progression of melanoma cell growth may be inhibited upon downregulation of both p38 MAPK and pERK1/2 [59], suggesting that tumor cell growth occurs as a balance between the contribution of different pathways exerting opposite metabolic effects.

## 4. Materials and Methods

### 4.1. Design and Synthesis of BS148 Fluorescent Probe

#### 4.1.1. Molecular Docking and Dynamics

The crystal structure of S2R in binary complex with roluperidone at 2.1 Å was retrieved from Protein Data Bank (PDB ID: 7M94) [13]. Chain A was prepared for calculations using VMD [60]. Missing bonds and atoms were fixed, and polar hydrogens and charges were added. Water molecules were removed. A three-dimensional grid box was centered on the oxygen atom of the hydroxyl group of Asp29. The center and the size of the grid maps were based on AutoGrid default values [size (x, y, z): 40/50/40 grid points spacing of 0.375 Å; center (x, y, z): 6.798/−6.705/16.560]. AutoDock 4.2.6 was used for the docking simulation [61]. Lamarckian genetic algorithm was employed for ligand conformational searching, and docking parameters were set as default. The docking model was validated first by redocking roluperidone into the prepared crystal structure with the resulting RMSD being within 0.95 Å. BS148-fluo was first drawn with ChemBio3D Ultra 14.0, and all the combinations of tautomers and protonation states at pH 7.4 of the ligands were generated with Discovery Studio. AutoDockTools 1.5.6 package scripts were used to convert structures of ligands and prepared protein to AutoDock 4.2.6 format. A total of 20 conformations with the best scores were retained. Lastly, docking poses were ranked according to the predicted scores and visually inspected with AutodockTools 1.5.6 and Discovery Studio software. GROMACS was used to simulate the docked pose of BS148-fluo in complex with S2R on an NVIDIA GeForce RTX 3070 Laptop GPU [62]. The S2R was embedded within a membrane bilayer. The positioning of S2R within the membrane was retrieved from the OPM (Orientation of Proteins in Membrane) database [63]. The complex was solvated using TIP3P water model and neutralized with Na^+^ counterions in a cubic box with at least 1 nm spacing from the ligand–protein complex. The complex was subjected to energy minimization using ‘‘steepest descent algorithm”. The complex was equilibrated for 100 ps using NVT followed by NPT ensemble. The MD production phase for all the systems was simulated for 50 ns with a time step of 2 fs and was performed in NPγT at 300 K and 1 bar. The production simulation with a total of 5000 frames was used for further MDS analysis. The root-mean-square fluctuations (RMSF) and root-mean square deviation (RMSD) were calculated using toolkits of the GROMACS package.

#### 4.1.2. Synthesis of BS148-Fluo

All the reagents and solvents were used as purchased without further purification. Air- or moisture-sensitive reactions were performed under nitrogen/argon atmosphere. Microwave reactions were performed with the microwave synthesizer Initiator (Biotage, Upsala, Sweden). The reactions were monitored using thin-layer chromatography on silica gel plates (60 Ǻ, F_254_, 70–230 mesh; Merk Life Science , Milan, Italy) and visualized with UV light or cerium ammonium sulfate solution. Column liquid chromatography (LC) purifications were carried out using Merck silica gel (60 Ǻ, 230–400 mesh, ASTM, Merck grade 9385; Merk Life Science, Milan, Italy). Flash chromatography purifications were performed using the Isolera One system (Biotage, Upsala, Sweden) and PLC was conducted (20 × 20 cm glass plate, silica gel 60 Ǻ, F_254_, 2 mm; Merk Life Science, Milan, Italy). The structures of all compounds were ensured using nuclear magnetic resonance (NMR) and mass spectrometry. ^1^H and ^13^C NMR (1D and 2D experiments) spectra were recorded on a DPX-400 Avance (Bruker, Billerica, MA, USA) spectrometer at 400 MHz or on an FT-NMR Avance III HD 600 (Bruker, Billerica, MA, USA) spectrometer at 600 MHz. Chemical shifts are expressed in ppm (δ). ^1^H NMR chemical shifts are relative to tetramethylsilane (TMS) as the internal standard. ^13^C NMR chemical shifts are relative to TMS at δ 0.0 or to the ^13^C signal of the solvent: CDCl_3_ δ 77.04, DMSO-d6 δ 39.5. NMR data are reported as follows: chemical shift, multiplicity (s, singlet; d, doublet; t, triplet; dd, double doublet; ddd, double double doublet; m, multiplet), coupling constants (Hz), and number of protons/carbons. The ^1^H and ^13^C NMR assignments are reported as follows: anbf, 4-amino-7-nitrobenzofurazane; bz, benzyl; dtsd, 1,4-dithiaspiro[4.5]decane; hexane, esano; ph, phenyl; pipd, piperidine. ^1^H–^1^H-correlation spectroscopy (COSY), ^1^H–^13^C heteronuclear multiple quantum coherence (HMQC), and heteronuclear multiple-bond connectivity (HMBC) experiments were recorded for the determination of ^1^H–^1^H and ^1^H–^13^C correlations. Melting points were determined using the Melting Point apparatus SMP30 (Cole-Parmer, Stuart, St. Neots, UK) and placing the samples in glass capillaries, and all the values are uncorrected. The purity of BS148-fluo was determined with elemental analysis (C, H, N), which was performed on a Carlo Erba 1106 Analyzer (Milano, Italy), and the results are within ±0.4% of the theoretical values. Low-resolution MS analysis was performed on an LC-MS^(n)^ Ion Trap 6310A (Agilent Technologies, Inc., Santa Clara, CA, USA), whereas high-resolution mass spectra were recorded on a UHPLC-HRMS 3000-Q Exactive (Thermo Fisher Scientific, Waltham, MA, USA), both equipped with an electrospray ionization source (ESI). The data here reported refer to the protonated molecular ion ([M+H]^+^).

NMR and LC-MS instruments were located and used at C.I.G.S.-UNIMORE (University of Modena, Italy).

*4-((1-((1,4-dithiaspiro[4.5]decane-2-yl)methyl)piperidin-4-yl)methyl)phenol* (**1**)

0.150 g (0.67 mmol) of 2-(chloromethyl)-1,4-dithiaspiro[4.5]decane was placed into a 0.5–2 mL microwave (MW) vial with 3 mL of anhydrous acetonitrile (can) and a catalytic amount of potassium iodide. Afterward, 0.307 g (1.35 mmol) of piperidin-4-ylmethylphenol and 0.143 g (1.35 mmol) of potassium carbonate were mixed in 2 mL of an. ACN and then added. The microwave reaction was carried out at 135 °C for 7 h total (two MW cycles of 3.5 h each). The solvent was evaporated under reduced pressure, and methylene chloride (MC) was added. The organic phase was washed with water, then with sodium carbonate saturated solution, and finally with brine, then it was separated, dried over sodium sulfate, filtered, and the solvent evaporated under reduced pressure. The crude (0.189 g) was purified with HPLC (high-performance liquid chromatography) using the Isolera One system (Biotage, Upsala, Sweden) and with a gradient elution: cyclohexane/ethyl acetate 95/5 → 90/10. The title compound was obtained in 14% yield (0.028 g, 0.095 mmol) as a colorless oil.

^1^H NMR (400 MHz, CDCl_3_): δ 1.34–1.45 (m, 2H, CH_2_-3/CH_2_-5 pipd), 1.39 (m, 2H, CH_2_-8 dtsd), 1.46 (m, 1H, CH-4 pipd), 1.54–1.69 (m, 2H, CH_2_-3/CH_2_-5 pipd), 1.61 (m, 4H, CH_2_-7 + CH_2_-9 dtsd), 1.95 (m, 2H, CH_2_-6/CH_2_-10 dtsd), 1.99 (m, 2H, CH_2_-6/CH_2_-10 dtsd), 2.05 (m, 1H, Ha CH_2_-6 pipd), 2.14 (m, 1H, Ha CH_2_-2 pipd), 2.43 (d, *J* = 6.6 Hz, 2H, CH_2_ bz), 2.55 (dd, *J* = 12.7, 6.0 Hz, 1H, Ha CH_2_N), 2.96 (dd, *J* = 12.7, 7.5 Hz, 1H, Hb CH_2_N), 3.04 (m, 1H, Hb CH_2_-2 pipd), 3.12 (m, 1H, Hb CH_2_-6 pipd), 3.19 (dd, *J* = 12.3, 5.9 Hz, 1H, Ha CH_2_-3 dtsd), 3.30 (dd, *J* = 12.3, 5.0 Hz, 1H, Hb CH_2_-3 dtsd), 3.95 (m, 1H, CH-2 dtsd), 6.75 (d, *J* = 7.7 Hz, 2H, CH-3 + CH-5 ph), 6.94 (d, *J* = 7.6 Hz, 2H, CH-2 + CH-6 ph). ^13^C NMR (100 MHz, CDCl_3_): δ 24.92 (C-8 dtsd), 25.68 (C-7/C-9 dtsd), 26.37 (C-7/C-9 dtsd), 31.06 (C-3/C-5 pipd), 31.16 (C-3/C-5 pipd), 37.53 (C-4 pipd), 41.00 (C-3 dtsd), 41.95 (CH_2_ bz), 42.82 (C-6/C-10 dtsd), 43.36 (C-6/C-10 dtsd), 51.80 (C-2 dtsd), 53.48 (C-6 pipd), 54.52 (C-2 pipd), 62.65 (CH_2_N), 68.65 (C-5 dtsd), 115.14 (C-3 + C-5 ph), 130.06 (C-2 + C-6 ph), 132.01 (C-1 ph), 154.21 (C-4 ph).

*4-((1-((1,4-dithiaspiro[4.5]decane-2-yl)methyl)piperidin-4-yl)methyl)phenyl-6-((7-nitrobenzo[c][1,2,5]oxadiazol-4-yl)amino)hexanoate (BS148-fluo)*

Into a 50 mL flask, a solution of 0.028 g (0.095 mmol) of 6-((7-nitrobenzo[c][1,2,5]oxadiazol-4-yl)amino)hexanoic acid in anhydrous MC was introduced, then 0.018 g (0.095 mmol) of 1-ethyl-3-(3-dimethylaminopropyl)carbodiimide (EDC) was added. The mixture was maintained under stirring and argon atmosphere for 5 min, then it was cooled (ice bath) for 1 h, and 0.018 g (0.048 mmol) of 4-((1-((1,4-dithiaspiro[4.5]decane-2-yl)methyl)piperidin-4-yl)methyl)phenol and triethylamine were added. The reaction was stirred at room temperature under argon atmosphere for 48 h. The mixture was taken up with MC and transferred into a separating funnel. The organic phase was washed with a saturated solution of sodium carbonate and ammonium chloride, then with water, and finally with brine. The organic phase was dried over sodium sulfate and filtered and the solvent removed under reduced pressure. The crude (0.030 g) was purified using column chromatography (room pressure) with 55/45 cyclohexane/ethyl acetate elution, giving 0.011 g (0.017 mmol, 35% yield) of the title compound as a very viscous, dark orange oil.

^1^H NMR (600 MHz, CDCl_3_): δ 1.39 (m, 2H, CH_2_-8 dtsd), 1.50–1.72 (m, 4H, CH_2_-7 dtsd + CH_2_-9 dtsd), 1.61 (m, 2H, CH_2_-4 hexane), 1.67 (m, 1H, CH-4 pipd), 1.76 (m, 2H, CH_2_-3/CH_2_-5 pipd), 1.81 (m, 2H, CH_2_-3/CH_2_-5 pipd), 1.84 (m, 2H, CH_2_-3 hexane), 1.88 (m, 2H, CH_2_-5 hexane), 1.93 (m, 2H, CH_2_-6/CH_2_-10 dtsd), 1.98 (m, 2H, CH_2_-6/CH_2_-10 dtsd), 2.57 (d, *J* = 6.9 Hz, 2H, CH_2_ bz), 2.60 (m, 1H, Ha CH_2_-6 pipd), 2.61 (t, *J* = 7.1 Hz, 2H, CH_2_-2 hexane), 2.70 (m, 1H, Ha CH_2_-2 pipd), 2.99 (dd, *J* = 13.3, 5.7 Hz, 1H, Ha CH_2_N), 3.28 (dd, *J* = 12.9, 4.2 Hz, 1H, Ha CH_2_-3 dtsd), 3.40 (dd, *J* = 12.9, 4.6 Hz, 1H, Hb CH_2_-3 dtsd), 3.55 (m, 2H, CH_2_-6 hexane), 3.62 (m, 1H, Hb CH_2_-2 pipd), 3.64 (dd, *J* = 13.3, 6.7 Hz, 1H, Hb CH_2_N), 3.81 (d, *J* = 12.1 Hz, 1H, Hb CH_2_-6 pipd), 4.15 (m, 1H, CH-2 dtsd), 6.18 (d, *J* = 8.7 Hz, 1H, CH-5 anbf), 6.43 (m, 1H, NH), 6.94 (dd, *J* = 8.4 Hz, 2H, CH-3 + CH-5 ph), 7.10 (dd, *J* = 8.4 Hz, 2H, CH-2 + CH-6 ph), 8.47 (d, *J* = 8.7 Hz, 1H, CH-6 anbf). ^13^C NMR (150 MHz, CDCl_3_): δ 24.21 (C-3 hexane), 24.74 (C-8 dtsd), 25.54 (C-7/C-9 dtsd), 26.20 (C-4 hexane), 26.54 (C-7/C-9 dtsd), 28.12 (C-5 hexane), 28.89 (C-3/C-5 pipd), 29.01 (C-3/C-5 pipd), 33.91 (C-2 hexane), 36.05 (C-4 pipd), 41.23 (C-3 dtsd), 41.33 (CH_2_ bz), 42.45 (C-6/C-10 dtsd), 43.00 (C-6/C-10 dtsd), 43.57 (C-6 hexane), 48.66 (C-2 dtsd), 52.99 (C-2/C-6 pipd), 55.14 (C-2/C-6 pipd), 62.60 (CH_2_N), 69.90 (C-5 dtsd), 98.59 (C-5 anbf), 121.42 (C-3 + C-5 ph), 123.97 (C-7 anbf), 129.93 (C-2 + C-6 ph), 136.49 (C-6 anbf), 136.58 (C-1 ph), 143.89 (C-4 + C-8 anbf), 144.28 (C-3 anbf), 149.08 (C-4 ph), 171.98 (C=O).

The free amine was then converted into oxalate salt. Additionally, 0.011 g (0.017 mmol) of BS148-fluo was dissolved in anhydrous diethyl ether (DE) and treated with oxalic acid (1 eq.). The mixture was left at room temperature under stirring for 24 h, then it was allowed to stand for 10 min. The obtained salt was recovered with filtration and washed three times with fresh an. DE to give 0.008 g (0.011 mmol, 63% yield) of an iridescent orange solid. HRMS–ESI: *m*/*z* [M+H]^+^ calcd for C_33_H_44_N_5_O_5_S_2_^+^: 654.2778, found: 654.2776 (dev. −0.41 ppm). Elemental analysis calcd for C_35_H_45_N_5_O_9_S_2_: C 56.51, H 6.10, N 9.41, found: C 56.69, H 6.10, N 9.38.

#### 4.1.3. Binding Assay of BS148-Fluo

Binding affinity at human S2R was performed with Eurofins using a radioligand displacement assay in the presence of [^3^H]-ditolylguanidine (at 25 nM) as nonselective S2R radioligand plus (+)pentazocine (at 1 μM) as selective S1R radioligand, as previously reported [64,65].

### 4.2. Cell Line Culture

SK-MEL-2 and MCF7 were cultured in Dulbecco’s Modified Eagle’s medium (DMEM, #L0106 Biowest, Nuaillé, France) supplemented with 10% fetal bovine serum (FBS, Gibco, Thermo Fisher Scientific, Waltham, MA, USA), 100 U/mL penicillin, 50 µg/mL streptomycin, and 2 mM L-glutamine (all from Sigma-Aldrich, a division of Merck KGaA, Darmstadt, Germany). Cells were maintained at 37 °C with 5% CO_2_ and humidified atmosphere in an incubator.

### 4.3. Confocal Microscopy Analysis

Before the confocal microscopy analysis, the different cell lines were grown to subconfluence on chamber slides overnight at 37 °C and 5% CO_2_. The next day, the cell culture medium was aspirated and the cells washed five times with PBS ten minutes each. The cell lines were incubated with 100 nM concentration of BS148-fluo 1 h at 37 °C. All cell lines on coverslip were washed 5 times with PBS, 10 min, and fixed in 4% paraformaldehyde (PFA) 15 min at 37 °C. To visualize the nuclei, samples were stained with DAPI 15 min at room temperature in the dark and re-washed. The coverslips were mounted with Mounting Medium in the center of the slide and sealed with a polish. The fluorescent intensity cells were examined using confocal microscopy.

### 4.4. Silencing of the Endogenous TMEM97 mRNA

Silencing of the endogenous *TMEM97* mRNA was performed using 150 nM of specific siRNA probes (#4392420; Thermo Fisher Scientific, Waltham, MA, USA) in cells transfected using the Lipofectamine 3000 reagent (Thermo Fisher Scientific, Waltham, MA, USA) following the manufacturer’s instructions. Negative control siRNAs were used as well (#4404021; Thermo Fisher Scientific, Waltham, MA, USA). Treatments were performed in 2 h serum-starved cells maintained 24 and 48 h in the presence of increasing BS148 concentrations (20–100 μM range). The cell viability was assessed with MTT assay, as previously described [66], and absorbance detected with a Victor3 plate reader (PerkinElmer Inc., Waltham, MA, USA).

### 4.5. Western Blot Analysis

The expression of S2R protein was assessed in lysates from SK-MEL-2 cells and PDX-derived cells (MM13, MM2, MM27, MM16) with Western blotting using a previously described protocol [67,68]. Firstly, the cells were seeded in 24-well plates (1 × 10^5^ cells/well) and they were lysed in ice-cold RIPA buffer. Then, the cell lysates were loaded onto 15% SDS-PAGE gel wells before electrophoresis. S2R protein expression was evaluated by incubating with a specific primary antibody (1:1000; #NBP1-30436, Novus Biologicals, part of the Bio-Techne srl, Milan, Italy). Signals were revealed with ECL chemiluminescent compound (GE HealthCare, Chicago, IL, USA) after incubation of membranes with a secondary anti-rabbit horseradish peroxidase (HRP)-conjugated antibody (#NA9340V; GE HealthCare, Chicago, IL, USA). β-actin (1:1000; #A3854, Sigma-Aldrich, a division of Merck KGaA, Darmstadt, Germany) was used as a protein loading control and detected using a mouse HRP-conjugated secondary antibody. pERK1/2 and p38 MAPK activation, γH2AX, and PARP-1 were evaluated in SK-MEL-2 treated 4 h with 40 µM and 80 µM BS148. Cells were lysed in ice-cold RIPA buffer containing protease and phosphatase inhibitors for protein extraction and loaded onto 12% SDS-PAGE gel wells. Total ERK (1:1000; #9102, Cell Signalling Technology Inc., Danvers, MA, USA) and tubulin (1:4000; #T6074, Sigma-Aldrich, a division of Merck KGaA, Darmstadt, Germany) were used as protein loading controls and specific primary antibodies for pERK1/2 and p38 MAPK (1:1000; #9101, #4511, respectively, Cell Signalling Technology Inc., Danvers, MA, USA), and γH2AX (1:1000; #05-636, Millipore, a division of Merck KGaA, Darmstadt, Germany) and PARP1 (1:1000; #sc-8007, Santa Cruz Biotechnology, Heidelberg, Germany) were used. Images were acquired using the Molecular Imager VersaDoc™ MP 4000 System and QuantityOne analysis software (Bio-Rad Laboratories srl, Segrate, Italy). Original images of western blots are provided in Supplementary Information (Figures S3 and S4).

### 4.6. RNA Extraction and qRT-PCR Analysis

RNA was purified and reverse-transcribed [69], then quantitative PCR was performed with SsoAdvanced Universal SYBR Green Supermix (#1725274, Bio-Rad Laboratories srl, Segrate, Italy) with the following oligonucleotides listed from 5′ to 3′: *TMEM97* (F:ttgcgagcttgtgtttcagc; R:ttgcaggagttcgaatccac), *RPS20* (F:gcgcctcttatcaagtcagc; R:cggaaaaacacccgtggag), *ACAT2* (F:cggcgcggaccatcatag; R:acccacactggcttgtctaa), *FDFT1* (F:tggactcgacagactctaagg; R:ttggtcaataagtcgcccacg), *FDPS* (F:agcctgttgtgtccgttttg; R:aggttcctctgtccacgctt), *ATF4* (F:gttctccagcgacaaggcta; R:atcctgcttgctgttgttgg), *CHOP* (F:cagaaccagcagaggtcaca; R:agctgtgccactttcctttc), *XBP1*s (F:tgctgagtccgcagcaggtg; R:gctggcaggctctggggaag), *BIP* (F:tgttcaaccaattatcagcaaactc; R:ttctgctgtatcctcttcaccagt). Data were analyzed using the Bio-Rad CFX Maestro 2.0 software (Bio-Rad Laboratories srl, Segrate, Italy), and mRNA expression was normalized to *RPS20* gene.

### 4.7. In Vitro Drug Treatment Viability and Migration Assay 

In vitro drug sensitivity was assessed with Cell Titer Glo Luminescent Cell Viability Assay (Cat#G7570; Promega, Madison, WI, USA), and cells were plated in 96-well plates (biological triplicate). PDX cells (2000 cells/well) were seeded and increasing concentrations of BS148 added 24h after cell seeding. Luminescence signal was assessed 24 h and 48 h afterward, and the relative viability (%) was calculated upon normalization to control, DMSO-treated cells. The migration assay was performed using inserts coated with fibronectin (5 μg/cm^2^, cat#11080938001 Roche) on the outer part of the filter in 24-well format (8.0-μm pore size, cat #353097; Corning Incorporated, Corning, NY, USA). PDX cells were pre-treated with BS148 (50 μM, 24 h) and then seeded for migration assay (70,000 MM2, 25,000 MM27, and 100,000 MM16 cells/Transwell) for 36h. Complete medium was added to the lower compartment of Transwells. Migrated cells were stained with 0.5% crystal violet solution (50% Crystal Violet 1% Sigma V-5265, 35% ethanol in water) after 24 h. Four images of each insert were acquired with an EVOS M5000 fluorescence microscope (Thermo Fisher Scientific, Waltham, MA, USA) and analyzed with ImageJ software to estimate occupied area and calculate migration rate (compared to control DMSO).

### 4.8. TCGA Data Analysis

Box plots of *TMEM97* expression were obtained from GEPIA [70], an interactive web server for analyzing the RNA sequencing expression data of tumors and normal samples from the TCGA and the GTEx projects using a standard processing pipeline (http://gepia.cancer-pku.cn/index.html, accessed on 6 March 2023). Kaplan–Meier analysis of overall survival in patients stratified according to high and medium/low expression of *TMEM97* and hazard ratio (HR) analysis were performed using OSskcm [71] (accessed on 6 March 2023), a survival analysis web server for skin cutaneous melanoma, using SKCM TCGA transcriptomic profiles.

### 4.9. Statistical Methods

Results from Cell Titer Glo cell viability assay were evaluated using one-way ANOVA, followed by Dunnett’s multiple comparison test, while MTT assay results were analyzed using two-way ANOVA. qRT-PCR results were analyzed using one-way ANOVA with Holm–Sidak’s multiple comparison test. Comparisons between two groups were performed with *t* test. Data were considered to be statistically significant if *p* <  0.05 (*), *p* <  0.01 (**), *p* <  0.001 (***), and *p* <  0.0001 (****). The statistical analysis results are reported in Supplementary Information Table S1.

## 5. Conclusions

In conclusion, in this work, we demonstrated that BS148-induced cytotoxicity is mediated by S2R targeting. BS148 binding to S2R decreases the expression of genes participating in the cholesterol signaling pathway also associated with the activation of the ER stress response, resulting in decreased proliferation of metastatic melanoma cells in vitro. Interestingly, BS148 treatment induces a decrease in S2R expression, producing the same effect as S2R RNA interference-mediated knockdown. In PDX models, BS148 reduces cell viability and migration properties, representing a promising preclinical candidate to be further developed for the treatment of metastatic melanoma.

## Figures and Tables

**Table 1:** Affinity constants (pK*i*) of BS148-fluo and the reference compound at S2R.

Compound	pK*i* S2R ^a^ ± SD
BS148-fluo	7.00 ± 0.11
BS148 *	7.71 ^b^

^a^ Values represent the mean of at least two separate experiments performed in duplicate. * See [18]. ^b^ SD is within ±20% [18].

## Data Availability

Not applicable.

## Supplementary Materials

The supporting information can be downloaded at: https://www.mdpi.com/article/10.3390/ijms24119684/s1.
