# Supplementary material for: BS148 Reduces the Aggressiveness of Metastatic Melanoma via Sigma-2 Receptor Targeting
Source: Int J Mol Sci. 2023 Jun 2;24(11):9684. doi: 10.3390/ijms24119684 (PMC10253401; doi:10.3390/ijms24119684)
Supplement: Supplementary file 1 [file ijms-24-09684-s001.zip › ijms-2327444-supplementary.pdf]

BS148 reduces the aggressiveness of metastatic melanoma via Sigma-2 receptor targeting

Claudia Sorbi,[1#] Silvia Belluti,[1#] Claudio Giacinto Atene,[2] Federica Marocchi,[3] Pasquale Linciano,[4] Neena Roy,[5] Elia Paradiso,[5] Livio Casarini,[5,6] Simone Ronsisvalle,[7] Tommaso Zanocco-Marani,[1] Livio Brasili,[1] Luisa Lanfrancone,[3] Carol Imbriano,[1] Giulia Di Rocco[1*] and Silvia Franchini[1*]

[1]*Department of Life Sciences, University of Modena and Reggio Emilia, Via Campi 287, 41125 Modena, Italy.*
[2]*Hematology Section, Department of Medical and Surgical Sciences, University of Modena and Reggio Emilia, 41124 Modena, Italy.*
[3]*Department of Experimental Oncology, IEO, European Institute of Oncology IRCCS, 20139 Milan, Italy.*
[4]*Department of Drug Sciences, University of Pavia, Pavia, Italy.*
[5]*Unit of Endocrinology, Department of Biomedical, Metabolic and Neural Sciences, University of Modena and Reggio Emilia, Ospedale di Baggiovara, via P. Giardini 1355, 41126, Modena, Italy.*
[6]*Center for Genomic Research, University of Modena and Reggio Emilia, Via G. Campi 287, 41125 Modena, Italy.*
[7]*Department of Drug and Health Sciences, Section of Medicinal Chemistry, University of Catania, 95125 Catania, Italy.*

Table of Content

Figure S1. Fig. 2 of main text - Molecular Dinamics simulation of BS148-fluo–protein complex. SI-2

Figure S2. Dose-response curve for BS148-fluo at S2R SI-3

Figure S3. Fig. 4B and 5B of main text - original images – Western Blot SI-4

Figure S4. Fig. 5C and 6A of main text - original images – Western Blot SI-5

Table S1. Fig. 4A, 4B, 5A and 6B of the main text - calculated p-values SI-6

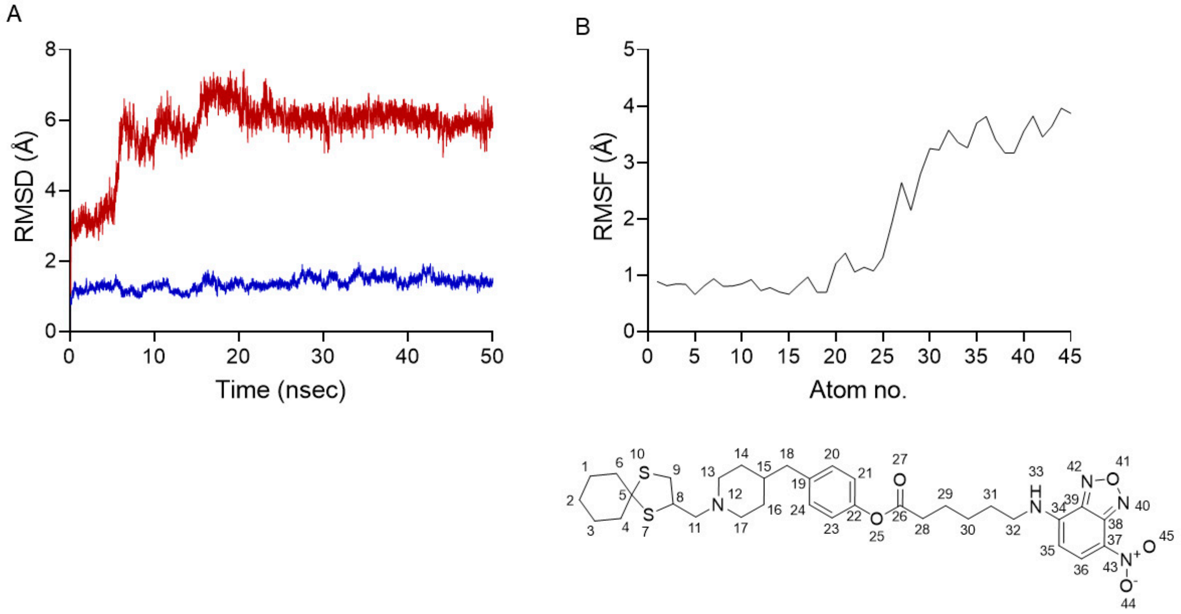

Figure S1. Fig. 2 of main text - Molecular Dinamics - (A) Backbone (blue line) and ligand (red line) RMSD over a period of 50 ns of MD simulation of BS148-fluo–protein complex. (B) RMSF values for BS148-fluo ligand; the atom numbering on the ligand structure is indicated.

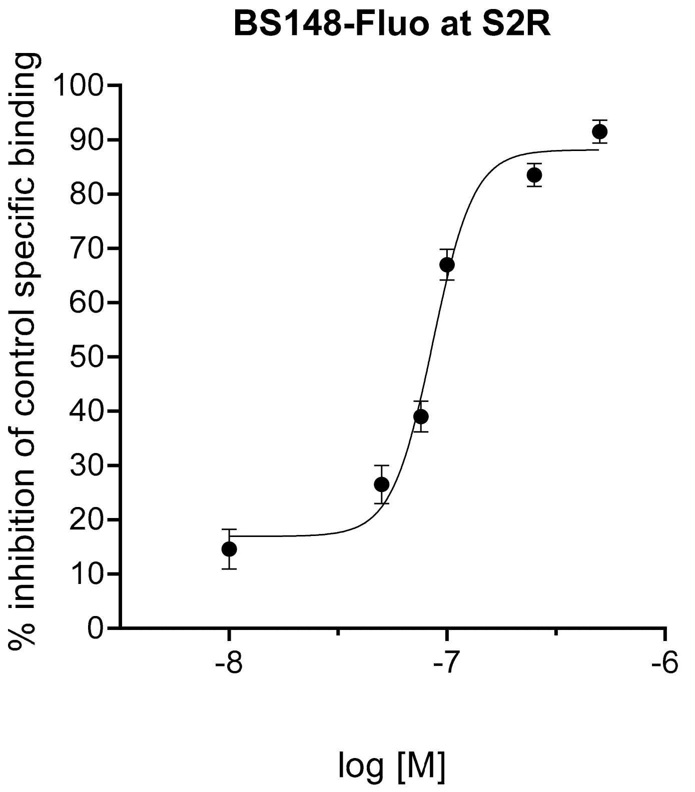

Figure S2. Dose-response curve for BS148-fluo at S2R.

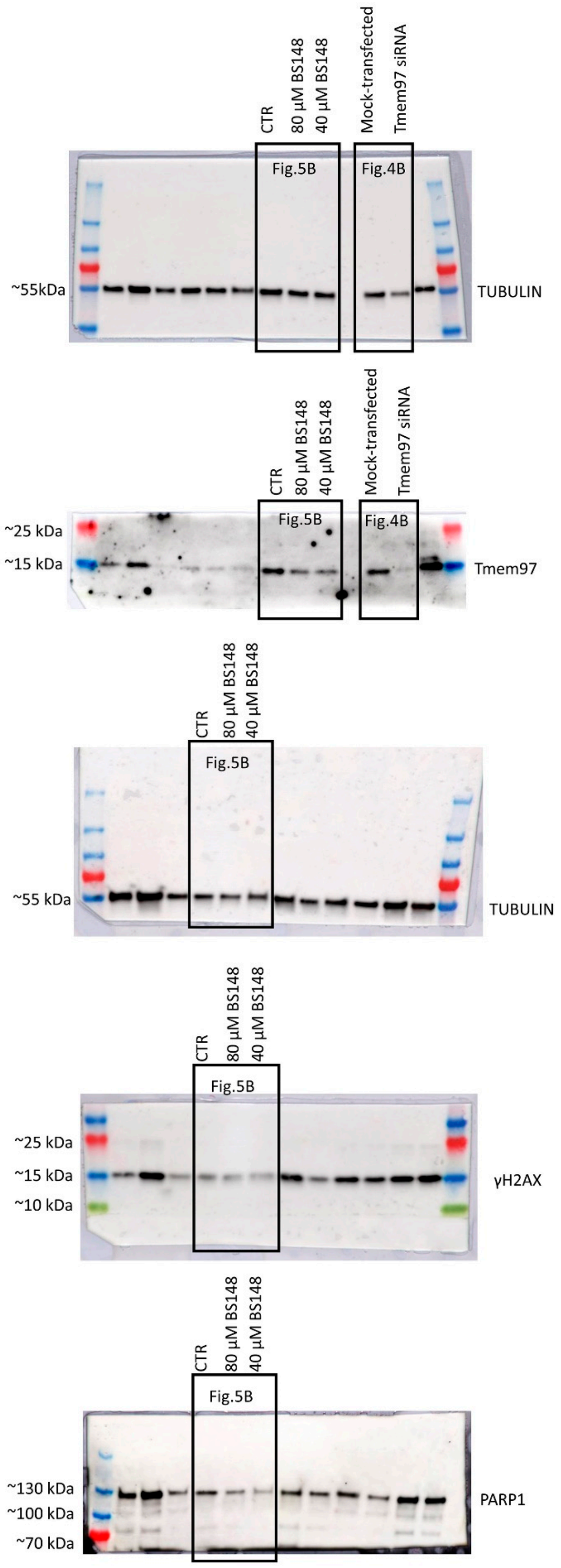

Figure S3. Fig. 4B and 5B of main text - original images – Western Blot

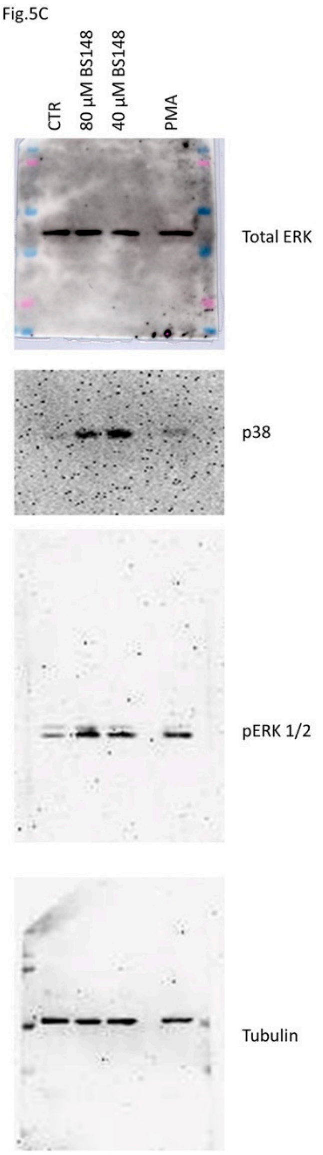

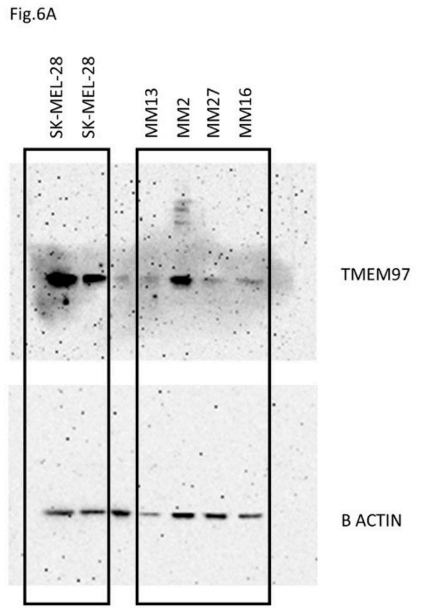

Figure S4. Fig. 5C and 6A of main text - original images – Western Blot

Figure 4A

t-test

Samples	Summary	P Value
SIGMA1R vs TMEM97	**	0.0062

Figure 4C

two-way ANOVA (multiple comparisons)

Samples	Summary	P Value
0 µM:siRNA mock vs. 60 µM:siRNA mock	**	0.0018
0 µM:siRNA mock vs. 80 µM:siRNA mock	***	0.0003
0 µM:siRNA mock vs. 100 µM:siRNA mock	***	0.0003
60 µM:siRNA mock vs. 60 µM:siRNA TMEM97	**	0.0047
80 µM:siRNA mock vs. 80 µM:siRNA TMEM97	*	0.0236
100 µM:siRNA mock vs. 100 µM:siRNA TMEM97	*	0.0239

Figure 5A

Ordinary one-way ANOVA (multiple comparisons)

	Samples	Summary	Adj. P Value
	40µM BS148 vs. CTR	**	0.0020
**TMEM97**	80µM BS148 vs. CTR	***	0.0007
	siTMEM97 vs. CTR	**	0.0025
	40µM BS148 vs. CTR	****	< 0.0001
**ACAT2**	80µM BS148 vs. CTR	****	< 0.0001
	siTMEM97 vs. CTR	****	< 0.0001
**FDFT1**	40µM BS148 vs. CTR	ns	0.2241
	80µM BS148 vs. CTR	ns	0.0633
	siTMEM97 vs. CTR	ns	0.2241
	40µM BS148 vs. CTR	**	0.0018
**FDPS**	80µM BS148 vs. CTR	***	0.0004
	siTMEM97 vs. CTR	**	0.0018
	40µM BS148 vs. CTR	ns	0.3863
**ATF4**	80µM BS148 vs. CTR	**	0.0023
	siTMEM97 vs. CTR	ns	0.3863
	40µM BS148 vs. CTR	ns	0.683
**CHOP**	80µM BS148 vs. CTR	***	0.0004
	siTMEM97 vs. CTR	*	0.0105
	40µM BS148 vs. CTR	ns	0.5878
**XBP1s**	80µM BS148 vs. CTR	*	0.0189
	siTMEM97 vs. CTR	*	0.0263
	40µM BS148 vs. CTR	ns	0.9797
**BIP**	80µM BS148 vs. CTR	ns	0.9674
	siTMEM97 vs. CTR	ns	0.9797

Figure 6B

Ordinary one-way ANOVA (multiple comparisons)

	24h		
	**Conc.(µM)**	**Summary**	**Adj. P Value**
**MM13**	0 vs. 40	*	0.0440
	0 vs. 80	**	0.0091
	0 vs. 100	**	0.0028
**MM27**	0 vs. 40	*	0.0151
	0 vs. 80	****	<0.0001
	0 vs. 100	****	<0.0001
**MM16**	0 vs. 40	ns	0.3092
	0 vs. 80	*	0.0310
	0 vs. 100	*	0.0127
**MM2**	0 vs. 40	ns	0.2197
	0 vs. 80	**	0.0024
	0 vs. 100	***	0.0002
	**48h**		
		**Summary**	**Adj. P Value**
**MM13**	0 vs. 40	ns	0.1424
	0 vs. 80	***	0.0008
	0 vs. 100	****	<0.0001
**MM27**	0 vs. 40	ns	0.1154
	0 vs. 80	**	0.0013
	0 vs. 100	***	0.0006
**MM16**	0 vs. 40	ns	0.9924
	0 vs. 80	***	0.0002
	0 vs. 100	****	<0.0001
**MM2**	0 vs. 40	**	0.0030
	0 vs. 80	****	<0.0001
	0 vs. 100	****	<0.0001

Table S1. Fig. 4A, 4C, 5A and 6B of the main text - calculated p-values (Prism software). (* $p < 0.05$; ** $p < 0.01$; *** $p < 0.001$; **** $p < 0.0001$; ns, not significant).
